# Premature Cardiac Contractions and Risk of Incident Ischemic Stroke

**DOI:** 10.1161/JAHA.112.002519

**Published:** 2012-10-25

**Authors:** Uchenna Ofoma, Fan He, Michele L. Shaffer, Gerard V. Naccarelli, Duanping Liao

**Affiliations:** Department of Critical Care Medicine, Mayo Clinic Rochester, MN (U.O.); Department of Public Health Sciences, Penn State College of Medicine, Hershey, PA (F.H., M.L.S., D.L.); Penn State Heart and Vascular Institute, Hershey, PA (G.V.N.)

**Keywords:** brain ischemia, embolic stroke, premature atrial contraction, premature ventricular contraction, risk factors

## Abstract

**Background:**

The etiologies of ischemic stroke remain undetermined in 15% to 40% of patients. Apart from atrial fibrillation, other arrhythmias are less well-characterized as risk factors. Premature cardiac contractions are known to confer long-term cardiovascular risks, like myocardial infarction. Ischemic stroke as cardiovascular risk outcome remains a topic of interest. We examined the prospective relationships in the Atherosclerosis Risk in Communities (ARIC) study, to determine whether premature atrial (PAC) or ventricular (PVC) contractions are associated with increased risk for incident ischemic stroke.

**Methods and Results:**

We analyzed 14 493 baseline stroke-free middle-aged individuals in the ARIC public-use data. The presence of PAC or PVC at baseline was assessed from 2-minute electrocardiogram. A physician-panel confirmed and classified all stroke cases. Average follow-up time was 13 years. Proportional hazards models assessed associations between premature contractions and incident stroke. PACs and PVCs were identified in 717 (4.9%) and 793 (5.5%) participants, respectively. In all, 509(3.5%) participants developed ischemic stroke. The hazard ratio (HR) (95% confidence interval [CI]) associated with PVC was 1.77 (1.30, 2.41), attenuated to 1.25 (0.91, 1.71) after adjusting for baseline stroke risk factors. The interaction between PVC and baseline hypertension was marginally significant (*P*=0.08). Among normotensives, having PVCs was associated with nearly 2-fold increase in the rate of incident ischemic stroke (HR 1.69; 95% CI 1.02, 2.78), adjusting for stroke risk factors. The adjusted risk of ischemic stroke associated with PACs was 1.30 (95% CI 0.92, 1.83).

**Conclusions:**

Presence of PVCs may indicate an increased risk of ischemic stroke, especially in normotensives. This risk approximates risk of stroke from being black, male, or obese in normotensives from this cohort.

## Introduction

Stroke is the leading cause of disability and the third-leading cause of death in the United States, with >700 000 incidents and >160 000 stroke-related deaths annually.^1^ Etiologies of ischemic stroke are well-documented but remain undetermined in 15% to 40% of patients.^2,3^ Numerous risk factors have been identified as targets of preventive strategies. Among cardiac risk factors, atrial fibrillation (AF) is the best-characterized.^4,5^ In all, 20% to 25% of ischemic strokes are from embolic complications of AF.^6,7^ The association between premature cardiac contractions and ischemic stroke is less well studied.

Premature ventricular contractions (PVCs) have been examined as predictors of cardiovascular morbidity and mortality,^8^ especially with pre-existing heart disease. The presence of PVCs was associated with a 2-fold increase in the rate of fatal coronary heart disease. Their role in ischemic stroke is less well studied but remains a topic of research interest.^9,10^

Premature atrial contractions (PACs) are known to precede AF^11,12^ and to be surrogate markers of paroxysmal AF in patients with acute ischemic stroke.^13,14^ PACs (but not PVCs) also were shown to be an independent risk factor for all strokes in a longitudinal study.^10^

The Atherosclerosis Risk in Communities (ARIC) Study^15^ is a prospective longitudinal, population-based study of atherosclerotic disease that provides a large cohort. Previous studies using this cohort showed that PVCs are associated with increased coronary heart disease (CHD) events and death.^8^ Another ARIC cohort study evaluating the association between metabolic syndrome clusters and ischemic stroke^16^ demonstrated a significant dose-response relationship between the number of metabolic syndrome components and risk of incident ischemic stroke. Metabolic syndrome also is known to be associated with subclinical cardiovascular disease^17^ and increased risk for incident cardiovascular disease.^18,19^ By extrapolation, it would appear that PVCs may confer some risk for ischemic stroke.

The objectives of this study therefore were to (1) evaluate the longitudinal relationship between premature cardiac contractions and incident ischemic stroke in a prospective general population-based cohort and (2) estimate the magnitude of PAC- and PVC-associated risk for incident ischemic stroke after controlling for prespecified cardiovascular risk factors.

## Methods and Population

The public-use ARIC study data were utilized for this analysis. ARIC, sponsored by the National Heart, Lung, and Blood Institute, is a population-based cohort study of atherosclerotic cardiovascular disease^15,20,21^ in 15 792 African Americans and whites, aged between 45 and 64 years, and randomly selected from 4 communities (Suburban Minneapolis, MN; Washington County, MD; Forsyth County, NC; and Jackson, MS). Baseline examination was conducted between 1987 and 1989. Cohorts were contacted yearly by telephone and followed-up by triennial clinical reexaminations. Follow-up data to 2002 were used in this report, with an average follow-up of 13 years. From the baseline sample we excluded 1299 individuals with baseline history of stroke or CHD, and 81 individuals who developed subarachnoid or intracerebral hemorrhage during follow-up, yielding an effective sample size of 14 493.

A supine resting 12-lead electrocardiogram (ECG) was obtained using the MAC PC10 personal cardiogram (Marquette Electronics, Milwaukee, WI) and a 2-minute, 3-lead (V1, II, and V5) rhythm strip. Participants were requested to fast and refrain from smoking and consuming caffeinated beverages before examination. Electrocardiographic procedures required the use of identified skin positions and placement of electrodes ≥3 minutes before recording. Electrocardiographic data processing, monitoring, and quality controls have been described elsewhere.^22^ Rhythm strips were classified 3 times by independent trained coders for total atrial ectopic beats, supraventricular, ventricular complexes and ventricular runs, bigeminy, trigeminy, and multiform complexes. Adjudication of disagreements was performed by the electrocardiographic center principal investigator or coding supervisor. PACs and PVCs were determined from the rhythm strip.

The identification of a stroke event was supported by an annual telephone follow-up and a community surveillance system in place to identify potential cardiovascular-related hospitalizations in the study-area hospitals. Using criteria adopted from the National Survey of Stroke, strokes were classified by computer algorithm and categorized into 1 of 4 main types: SAH, intracerebral hemorrhage (ICH), thrombotic brain infarction, or embolic brain infarction.

Medical records from all potential cardiovascular-disease-events-related hospitalizations and all death certificates were obtained and abstracted. The involved health care providers and next-of-kin were interviewed to obtain symptoms, time of symptoms, and any other stroke-relevant findings not included in hospital records. Relevant data for stroke-event classification included the sudden onset of neurological symptoms lasting for more than 24 hours or leading to death, the type and duration of patients' initial neurological symptoms, medical history, results of medical procedures, medications, reports from imaging (CT or MRI), autopsy findings, and other supportive clinical evidence.

In addition to a computer-determined diagnosis, cases were independently reviewed by a physician. The final diagnosis was determined by agreement of computer and reviewer classification. Disagreements were adjudicated by a second physician-reviewer.^20,21^

The following covariates known to be associated with premature cardiac contractions and stroke, and ascertained during baseline examination, were considered as major confounders: age, gender, race, body mass index (BMI), hypertension, diabetes, smoking, and total cholesterol levels. Hypertension was defined as an average of the final 2 blood pressure measurements of diastolic ≥90 mm Hg or systolic ≥140 mm Hg or self-reported use of medications for hypertension. Participants were classified as having diabetes if the fasting glucose level was ≥7 mmol/L (126 mg/dL). BMI was modeled as a continuous variable, and as a 3-level categorical variable using current standard definitions of overweight and obesity used by the Centers for Disease Control (CDC).^23^ Cholesterol also was modeled as a continuous variable (total cholesterol) and a 2-level categorical variable denoting high cholesterol levels or otherwise. High cholesterol was defined as total cholesterol ≥5.7 mmol/L (220 mg/dL), or the use of lipid-lowering medications. Prevalent CHD at baseline was determined by a history of myocardial infarction, heart or arterial revascularization surgery (coronary bypass or coronary angioplasty), or evidence of myocardial infarction on an adjudicated ECG at the ARIC baseline examination.

The study sample characteristics were summarized as means (SD) or proportions, stratified according to ectopy status. Cox proportional hazards regression models^24^ were used to compute the hazard ratios (HR) of ischemic stroke, comparing different ectopy types. Ischemic stroke included both types of infarctions (thrombotic and embolic brain infarction). Age, race, gender, and hypertension status were tested as potential effect-modifiers. All statistical analyses were performed using SAS 9.1 (SAS Institute Inc, Cary, NC).

## Results

PACs were demonstrated on baseline 2-minute ECG in 717 (4.9%), while PVCs were identified in 793 (5.5%) of the stroke and CHD-free baseline cohort. Among persons with baseline PVC on 2-minute ECG, 42% had 1 PVC (30 PVCs/hour), 16% had 2 PVCs (60 PVCs/hour), 10% had 3 PVCs (90 PVC/hour), and 32% had 4 or more PVCs (120 PVCs or more/hour). Five hundred and nine (3.5%) individuals developed ischemic stroke during 13 years of follow-up. Specifically, 39 (5.4%) of the subjects with PACs and 44 (5.6%) with PVCs on baseline ECGs developed ischemic strokes. The baseline characteristics of study participants are summarized in Table 1. While groups with and without PVCs differed significantly with respect to age, gender, race, BMI, hypertension, and diabetes, groups with and without PACs differed significantly only with respect to age and cigarette smoking.

**Table 1.** Demographic Data, Baseline Characteristics, and Incident Stroke Rates

		PVC			PAC		
			
Characteristic	N=14 493	Yes=793	No=13 700	*P*	Yes=717	No=13 776	*P*
Age, years	53.96	55.97	53.85	<0.0001	56.14	53.85	<0.0001

Gender, %							

Male	43.28	49.18	42.94	0.001	44.35	43.23	0.55
			
Female	56.72	50.82	57.06		55.65	56.77	

Race, %							

Non-white	26.91	31.90	26.62	0.001	26.22	26.95	0.67
		
White	73.09	68.1	73.38		78.78	73.05	

BMI, kg/m^2^	27.66	28.47	27.61	<0.0001	27.43	27.67	0.28

Total cholesterol, mg/dL	214.54	211.85	214.69	0.07	214.12	214.56	0.79

Hypertension, %	33.44	44.54	32.80	<0.0001	35.86	33.32	0.16

Diabetes, %	10.93	13.98	10.76	0.005	9.42	11.01	0.19

Smoking, %	25.83	25.73	25.83	0.95	29.85	25.62	0.01

Incident stroke, %	3.51	5.55	3.39	0.001	5.44	3.41	0.004

PVC indicates premature ventricular contractions; PAC, premature atrial contractions; and BMI, body mass index.

Individuals with PVCs and PACs had nearly double the rate of ischemic stroke in comparison to subjects without any form of ectopy (Model A, Table 2). When adjusted for age, race, gender, BMI, total cholesterol, diabetes, hypertension, and cigarette smoking, the risk is attenuated to 1.25 and 1.30 for PVC and PAC, respectively (Model B, Table 2). This model also takes into consideration the possibility of simultaneous presence of both PACs and PVCs by adjusting for one, when the other was the outcome.

**Table 2.** Multivariable Adjusted Risk of Ischemic Stroke in Relation to PAC and PVC

	Model A		Model B	
		
	HR	95% CI	HR	95% CI
PVC or PAC	1.73	(1.36, 2.20)	1.25	(0.97, 1.61)

PAC	1.68	(1.21, 2.32)	1.30	(0.92, 1.83)

PVC	1.77	(1.30, 2.41)	1.25	(0.91, 1.71)

HR indicates hazard ratio; PVC, premature ventricular contractions; and PAC, premature atrial contractions.

A: Unadjusted HR and 95% CI for PVC or PAC in relationship to incident ischemic stroke.

B: Adjusted for age, race, gender, BMI, total cholesterol, diabetes, hypertension, smoking, PAC (PVC as predictor), and PVC (PAC as predictor).

Hypertension was a marginally significant effect-modifier for the PVC-ischemic stroke relationship (*P*=0.08 for the interaction term), but not for the PAC-ischemic stroke relationship (*P*=0.76). Therefore, we performed exploratory stratified analysis on the relationship between PVC and ischemic stroke according to baseline hypertension status. The increased rate of ischemic stroke associated with PVCs persisted among normotensives after controlling for traditional stroke risk factors; HR 1.69 (95% CI 1.02 to 2.78). However, the association between PVC and ischemic stroke among baseline hypertensives was not statistically significant (HR 1.07, 95% CI 0.71, 1.62). To elucidate the risk associated with PVC as compared to other stroke risk factors, we included PVC and other dichotomized risk factors in one model among baseline normotensives. These results are shown in Table 3, where the observed risk associated with PVC was comparable in magnitude to the stroke risk attributable to being obese, black, or male and slightly lower than smoking risk. Exploratory analyses adjusting for incident myocardial infarction and incident hemorrhagic stroke as time-dependent covariates did not alter the relationship between PVCs and ischemic stroke among normotensives or hypertensives. (Supplemental Table).

**Table 3.** Multivariable Adjusted Risk of Ischemic Stroke in Relation to PVC and Covariates

	Adjusted HR (95% CI)	
	
Variable	Normotensive	Hypertensive
Age, years	1.11 (1.08, 1.14)	1.06 (1.04, 1.08)

Race (black vs white)	1.63 (1.17, 2.27)	1.66 (1.32, 2.10)

Gender (male vs female)	1.66 (1.24, 2.23)	1.53 (1.22, 1.93)

High cholesterol*	1.03 (0.77, 1.37)	1.20 (0.90, 1.59)

Obese vs Normal weight†	1.61 (1.09, 2.40)	1.16 (0.83, 1.62)

Overweight vs Normal weight†	1.29 (0.91, 1.83)	1.17 (0.85, 1.62)

Diabetes	2.97 (2.07, 4.26)	2.73 (2.15, 3.48)

Smoking	1.95 (1.45, 2.63)	2.06 (1.61, 2.63)

PVC	1.69 (1.02, 2.79)	0.97 (0.64, 1.48)

HR indicates hazard ratio; PVC, premature ventricular contractions.

* High cholesterol defined as total cholesterol ≥220 mg/dL or the use of lipid-lowering medications.

† Normal weight=BMI 18 to 24; Overweight=BMI 25 to 29; Obese=BMI ≥30.

## Discussion

In this population-based cohort, we found increased risks for ischemic stroke among subjects with premature cardiac contraction beats in models not adjusted for traditional stroke risk factors. However, after adjusting for age, gender, and other stroke risk factors, the hazard ratios were largely attenuated, and the 95% CI included one in both cases. In a baseline-hypertension-status stratified model, we further found that among individuals without hypertension, having PVCs resulted in a substantially higher rate of ischemic stroke compared with those without PVCs. This risk remained statistically significant after adjusting for well-established stroke risk factors. Our observed PVC effect -size for rate of ischemic stroke (HR 1.69) in baseline normotensives free of stroke and CHD approaches the PVC effect -size for rate of fatal CHD (HR 2.14) and death (HR 1.48) in a previous report.^8^ In comparison to the adjusted risks for other covariates, having PVCs carried similar risk of stroke among normotensives as being black (HR 1.63), male (HR 1.66) or obese (HR 1.61). PVC only ranks lower than smoking (HR 1.95) and diabetes (HR 2.97).

There are no prior reports of any positive longitudinal relationship between PVCs and pure ischemic stroke in any patient populations. In a Swedish male cohort study,^10^ Engstrom et al found no significant relationship between PVCs and all-cause stroke (ischemic, hemorrhagic and nonspecified stroke). The prevalence of PVCs (Lown and Wolf class 2 to 5^25^) was 36%, compared to 5.5% in our study. Differences in study population, detection method, duration of observation and studied outcomes may explain the discrepancy in prevalence rates and statistical significance. The Swedish cohort was comprised entirely of men (in whom PVCs are more frequent); only excluded subjects with baseline history of myocardial infarction, while our study excluded patients with baseline history of CHD (in whom PVCs are also more common). Also, 24-hour ambulatory ECG is more sensitive than the 2-minute ECG of the ARIC study. However, considering that the prevalence of more frequent or complex PVCs in the ARIC study population was 3% and 0.8%, respectively,^26^ the Swedish-study prevalence is quite high and inexplicable.

More recently, PVCs were shown to be independently associated with incident stroke (ischemic and hemorrhagic) among individuals without hypertension and diabetes at baseline.^9^ While this study demonstrated a stronger association for embolic stroke than for thrombotic stroke, it did not specifically examine the relationship between PVCs and pure ischemic stroke.

Hypertension with or without ventricular hypertrophy is known to increase the prevalence of PVCs.^27,28^ In the same ARIC cohort, hypertension was shown to be associated with a 23% increase in PVC prevalence.^26^ By extension, this should result in a stronger association between PVC and stroke among hypertensives. However, our data suggest otherwise. Pathophysiologic mechanisms through which PVCs lead to adverse cardiovascular outcomes like stroke and sudden cardiac death are not well understood. Therefore, while the clinical correlation of our finding is not entirely explicable, it is likely that PVCs in normotensives represents the overall burden of other risk factors, especially those not adjusted for in the model or those that may have not have been present at baseline but may have developed during follow-up, for example, latent CHD. However, adjustment for interim myocardial infarction did not significantly alter the PVC effect. The similarity of our findings compared to that of Massing et al supports this conclusion. However, the exact reasons for the lack of PVC and stroke association among hypertensives are unclear. Epidemiological data like those presented here may be insufficient to identify such mechanistic links. It is possible that among hypertensives, other important risk factors, such as those included in the models to generate the results in Table 3, are competing for the variance explained by PVC, leading to diminished PVC -effect. In other words, it is arguable that PVCs in hypertensive subjects may not be a better marker of overall burden of cardiovascular risk than other traditional stroke risk factors.

PACs have been retrospectively shown to be surrogate markers of AF in patients with acute ischemic stroke,^13,14^ but our study failed to show a significant longitudinal relationship between PAC and ischemic stroke. Again, this contrasts with the Swedish study which found that a high frequency of PACs was significantly associated with increased stroke risk, independent of other major risk factors. The prevalence of PACs in that study was 19% compared to 4.9% in our study, while the stroke rate was 14% compared to 3.5% in our study. The frequency and prevalence of PACs are known to increase with age.^29^ As with PVCs, differences in study populations (older male), detection methods (24-hour monitoring) and outcomes (composite stroke) may explain this discrepancy.

One shortcoming of our study is that it is unclear how many subjects developed AF during follow-up. The ARIC study performed ECGs at baseline only. The observed stroke risk may therefore be due to undetected AF rather than a direct effect of premature cardiac contractions. Potential for misclassification also exists from systematic under-diagnosis of premature contractions based on 2-minute ECGs as opposed to 24 hour monitoring. Mere presence of PVCs on 2-minute ECG is an insensitive predictor of PVCs based on 24 hour monitoring.^30^ Baseline prevalence of premature contractions as well as effect sizes may therefore be greater than observed. PACs may therefore actually pose a higher and perhaps significant stroke risk, while PVCs may pose much greater risk than observed in normotensives. While these finding are very preliminary, additional studies using more sensitive instruments to study the risk of pure ischemic stroke in a general population may be warranted. Additionally, the use of antiarrhythmics was not factored into our analysis. While this is a potential limitation, previous studies on implication of the use of beta-blockers, calcium channel blockers and antiarrhythmics as they relate to cardiovascular events in the ARIC cohort^7^ showed that PVCs were associated with increased risk for future cardiovascular events despite multivariate adjustments for use of these medications.

Any findings of significant association between premature cardiac contractions and ischemic stroke pose fundamental clinical concern as it relates to how such potential risks should be mitigated against. Although we demonstrated an increased stroke rate associated with PVC, such observational data cannot be interpreted in the context that eliminating PVCs pharmacologically would eliminate the risk. Antiarrhythmic drugs have been shown to suppress PVCs but increase the risk of death from CHD,^31,32^ and are no longer routinely used for this purpose. While there is little evidence that risk factor control decreases the prevalence of PVCs, they do decrease the risk of strokes and may decrease the risk of cardiac events.^33–35^ The finding of PVCs on routine electrocardiography in apparently healthy patients may suggest the need to search for and treat modifiable cardiovascular risk factors, especially in normotensives.
